# Objective Assessment of Postoperative Morbidity After Breast Cancer Treatments with Wearable Activity Monitors: The “BRACELET” Study

**DOI:** 10.1245/s10434-021-10458-4

**Published:** 2021-07-26

**Authors:** Nur Amalina Che Bakri, Richard M. Kwasnicki, Kieran Dhillon, Naairah Khan, Omar Ghandour, Alexander Cairns, Ara Darzi, Daniel R. Leff

**Affiliations:** 1 Department of Surgery and Cancer, Imperial College London, London, UK; 2 Academic Surgical Unit, Imperial College Healthcare NHS Trust, St. Mary’s Hospital, London, UK

## Abstract

**Background:**

Current validated tools to measure upper limb dysfunction after breast cancer treatment, such as questionnaires, are prone to recall bias and do not enable comparisons between patients. This study aimed to test the feasibility of wearable activity monitors (WAMs) for achieving a continuous, objective assessment of functional recovery by measuring peri-operative physical activity (PA).

**Methods:**

A prospective, single-center, non-randomized, observational study was conducted. Patients undergoing breast and axillary surgery were invited to wear WAMs on both wrists in the peri-operative period and then complete upper limb function (DASH) and quality-of-life (EQ-5D-5L) questionnaires. Statistical analyses were performed to determine the construct validity and concurrent validity of WAMs.

**Results:**

The analysis included 39 patients with a mean age of 55 ± 13.2 years. Regain of function on the surgically treated side was observed to be an increase of arm activity as a percentage of preoperative levels, with the greatest increase observed between the postoperative days 1 and 2. The PA was significantly greater on the side not treated by surgery than on the surgically treated side after week 1 (mean PA, 75.8% vs. 62.3%; *p* < 0.0005) and week 2 (mean PA, 91.6% vs. 77.4%; *p* < 0.005). Subgroup analyses showed differences in recovery trends between different surgical procedures. Concurrent validity was demonstrated by a significant negative moderate correlation between the PA and DASH questionnaires (*R* = −0.506; *p* < 0.05).

**Conclusion:**

This study demonstrated the feasibility and validity of WAMs to objectively measure postoperative recovery of upper limb function after breast surgery, providing a starting point for personalized rehabilitation through early detection of upper limb physical morbidity.

**Supplementary Information:**

The online version contains supplementary material available at 10.1245/s10434-021-10458-4.

Breast cancer is the most commonly diagnosed cancer worldwide, with estimated diagnoses of 2.3 million new cases in 2020, representing 11.7% of all the cancer cases in the world.^1^ During recent decades, the prognosis of breast cancer has significantly improved such that the current 5- and 10-year survival rates after breast cancer treatments in countries with advanced medical care are approximately 90% and 80%, respectively.^2^,^3^

Breast cancer treatments often are accompanied by long-term morbidity, which can have a severe impact on a patient’s quality of life (QoL) due to pain, reduced range of movement, and lymphedema.^4^–^11^ Given the high prevalence breast cancer and improved survival rates, the impact of treatments on breast cancer survivors should be objectively documented.^12^

Surgery remains the mainstay of curative treatment for breast cancer.^13^ Breast and axillary surgery have evolved from radical mastectomies to the breast-conserving approach of partial mastectomy and skin-sparing mastectomies combined with reconstruction in an attempt to minimize iatrogenic morbidity and improve QoL.^14^ Similarly, evolution in axillary staging procedures with the introduction of sentinel lymph node biopsy (SLNB) and the modern era of “axillary de-escalation,”^15^,^16^ which seeks to minimize rates of axillary lymph node dissection (ALND), are motivated by maximizing oncologic outcomes while minimizing upper limb morbidity.

The misconception that disabilities resolve over time without intervention and that “axillary de-escalation” has eliminated the physical morbidity of breast cancer surgery has arguably led to a reduced emphasis on monitoring disabilities, especially by objective means.^17^ Although various methods to capture postoperative morbidity have been described, the most common outcome measures used clinically and in research are QoL questionnaires and arm volume measurements.^18^,^19^ These may be useful, but they may not reliably capture the functional morbidity and may provide poor comparisons between patients.^20^ Moreover, questionnaires are prone to recall bias.^21^,^22^

Sensing technologies enable the acquisition of functional data that may provide new insights into postoperative recovery and facilitate personalized interventions.^23^ Wearable activity monitors (WAMs) enable arm movements to be captured during the peri-operative period in a non-obtrusive manner. They are increasingly used to measure the physical activity of patients with cancer, stroke, and Parkinson’s disease, and in orthopedic surgery.^24^–^27^ Their use to measure, monitor, and provide feedback on levels of physical activity to enhance recovery are motivated by improvements in outcomes and QoL.^21^,^28^,^29^

The majority of trials using WAMs in breast cancer survivors have focused on step counts and general daily activities.^24^ To the best of our knowledge, objective assessment of functional recovery specifically monitoring upper limb activities after breast or axillary surgery using WAMs has not been conducted previously. This observational study aimed to assess the feasibility of using WAMs to measure the arms’ functional recovery after breast and axillary surgery.

## Methods

### Design

A single-center, prospective, non-randomized, observational cohort study at Imperial College Healthcare NHS Trust was designed to characterize upper limb functional recovery after breast and axillary surgery using WAMs. Ethical approval was granted by the National Research Ethics Committee (ref. 15/LO/1038), and the study methods were submitted to the ClinicalTrial.gov registry (NCT03635723). All participants recruited provided informed written consent. The National Institute of Health Research (NIHR) Imperial Biomedical Research Centre provided funding through competitive application in 2018.

### Protocol

Patients undergoing breast surgery with or without axillary surgery were identified and assessed for eligibility in outpatient clinics and multi-disciplinary team meetings from April 2019 to December 2020. Eligible patients were invited to participate in the study. If interested, they had the study fully explained to them verbally, and a patient information sheet (PIS) was provided. Patient demographics were documented and included the medical records number (MRN), date of birth, type of operation (partial mastectomy, skin-sparing mastectomy, SLNB, ALND and immediate breast reconstruction (IBR)), and date of surgery.

A checklist was completed for all the patients to ensure compliance with NIHR Good Clinical Practice. The patients completed pre- and postoperative Disability of the Arm, Shoulder, and Hand (DASH) and EuroQol-5D-5L (EQ-5D-5L) questionnaires. The DASH questionnaire has 30 items focused on upper extremity ability, with a score ranging from 0 to 100 and with a lower score reflecting minimal disability.^30^,^31^ The EQ-5D-5L questionnaire is a validated instrument containing a descriptive component and a visual analogue scale (VAS) that can be used in a wide array of health conditions for measuring health-related QoL.^32^,^33^

### Inclusion and Exclusion Criteria

Patients undergoing any form of oncologic or reconstructive breast surgery with or without axillary surgery including partial mastectomy, mastectomy, implant or autologous reconstruction, ALND, or SLNB were eligible for recruitment. There was no restriction placed on age. Patients who had a movement disorder (e.g., Parkinson’s disease), those using a mobility device or aids, and those with inadequate comprehension (after attempted translation/explanation via staff or family members) were excluded from the study (*n* = 5).

Three patients with benign histology also were included in the study because the purpose of the WAMs is to assess the effect from the management of the disease (i.e., iatrogenic effect) rather than the type of disease. Phyllodes tumor was diagnosed for two of the patients, who had a partial mastectomy. The final histology from the surgery showed benign tumor. The remaining patient had a breast biopsy, which showed papilloma with ductal carcinoma *in situ* (DCIS). She underwent left mastectomy with deep inferior epigastric perforators (DIEP) flap reconstruction. The final histology from the surgery showed florid papillomatosis and focal atypia.

### Procedures

**Fig. 1:**
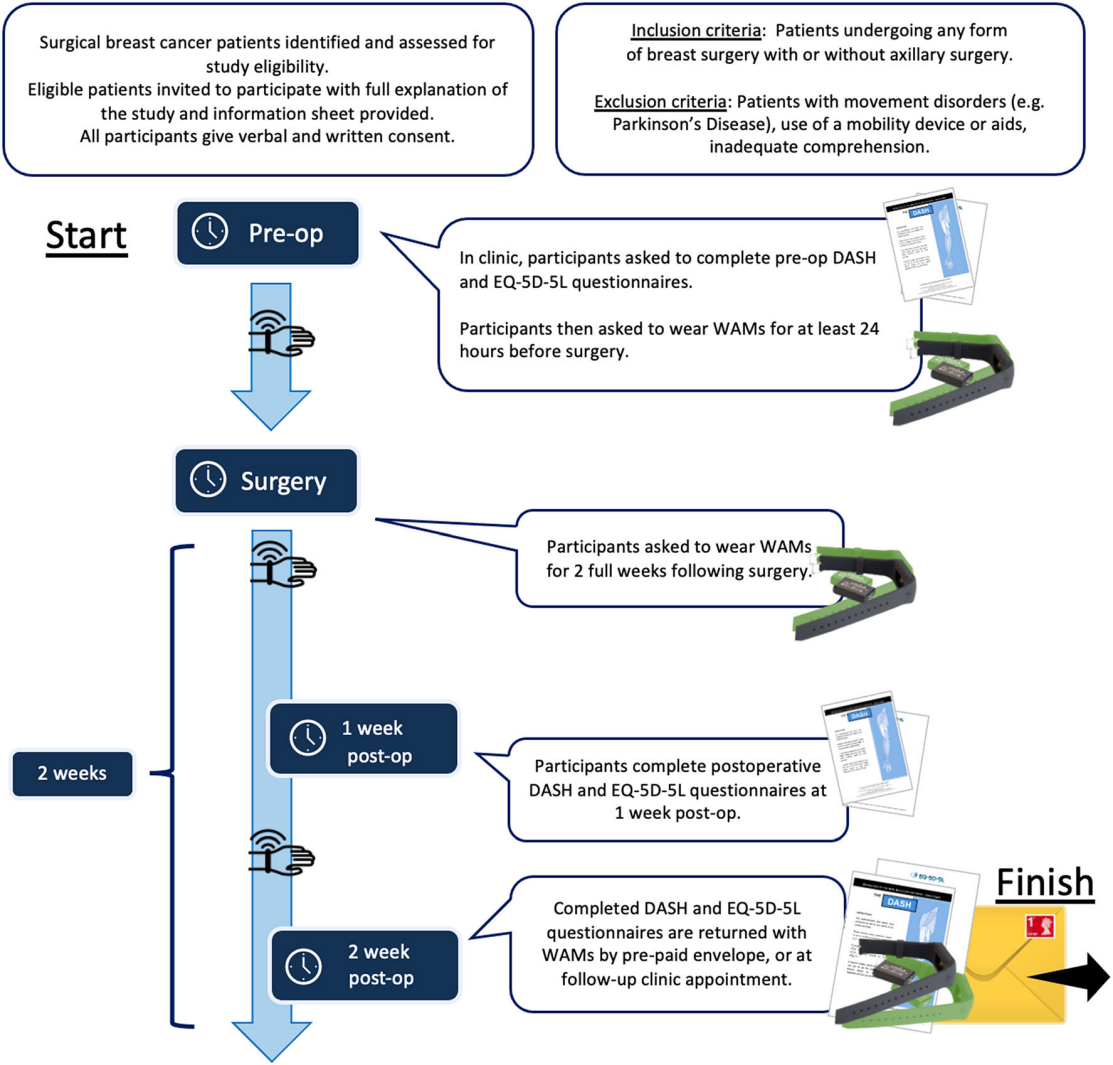
Recruitment protocol as well as inclusion and exclusion criteria. DASH, disability of the arm, shoulder, and hand questionnaire; EQ-5D-5L, EuroQol-5D-5L questionnaire; WAMs, wearable activity monitors

The patients wore WAMs on both wrists daily for at least 24 h before the operation and again daily for as long as 2 weeks postoperatively (Fig. 1). They were advised to wear the WAMs 24 h a day but were permitted to remove them during sleep and bathing if required. The patients were informed that the sensors measured arm movements but were not given any activity goals. However, it is standard practice in our unit to give all patients standard postoperative arm mobility recommendations as well as a leaflet on postoperative exercises from Breast Cancer Care. The leaflet can be found on www.breastcancernow.org, and all the postoperative exercise instructions can be found in the leaflet.

The patients were advised to begin mobilizing their arms the day after their surgery when possible and to continue doing so in a graduated manner until they were back to their normal range of movement before surgery. They were advised to stop doing the exercise or speak to a clinician as soon as possible if they had seroma, wound infection/healing problems, or pain that worsened during these exercises or continued once they had finished them.

**Fig. 2:**
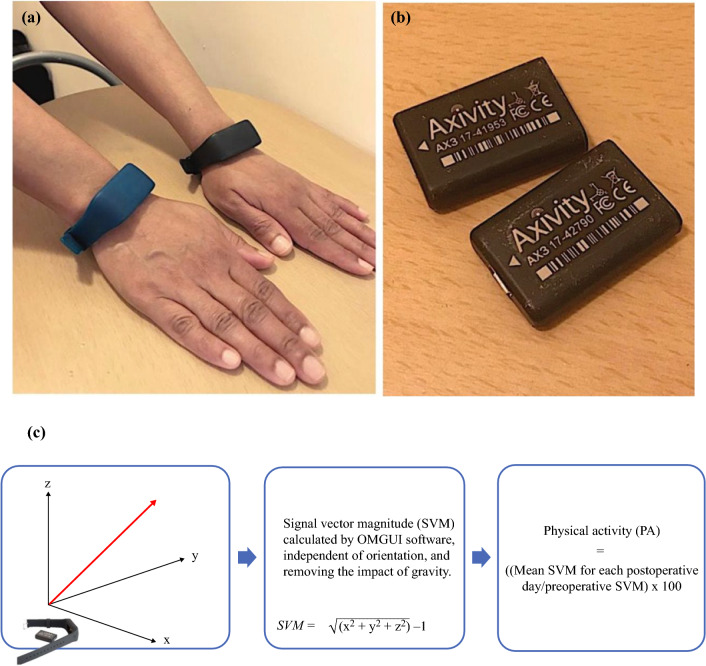
**a** Wearable activity monitors (Axivity AX3, designed by Open Lab, Newcastle University) worn on both wrists. The AX3 sensors were placed in the bracelets (green for right arm and black for left arm). **b** Axivity AX3 logging accelerometer devices when removed from the bracelets. **c** Signal vector magnitude (SVM) is the magnitude of acceleration in three directions (x, y, and z) giving a measure for activity of the upper limb movements. SVM is calculated by OMGUI software using the equation, SVM-1 = sqrt(x^2^ + y^2^ + z^2^) – 1. Physical activity (PA), defined by the percentage of the preoperative level, was calculated for the right and left arms in the patient cohort (mean SVM for each postoperative day/preoperative SVM × 100).

The questionnaires and sensors were returned by prepaid envelope after the postoperative period or at the follow-up clinic appointment. The wrist-worn sensors (AX3; Axivity, Newcastle upon Tyne, UK^34^) are commercially available triaxial accelerometers (Fig. 2) that allow manual calibration (e.g., data capture frequency and sensitivity), full data download, and analysis in bespoke software. Wrist-worn triaxial accelerometers had been validated to measure upper limb activities in previous studies.^35^–^37^ The Open Movement Graphical User Interface OMGUI (version 1.0.0.37) was used for calibration and calculations of activity levels using signal vector magnitude (SVM),^34^,^38^ which is a well-established method of combining triaxial accelerometer data to provide a quantitative activity level. All the patients were interviewed after (or during) the data collection period so they could state any technical issues encountered or report if the WAM had been removed for any period. Any lost or broken WAMs were replaced (*n* = 8).

Preoperative sensor data during the 24 h before the operation date were used as a baseline to assess postoperative upper limb activity. Physical activity (PA), defined by the percentage of the preoperative level (Fig. 2), was calculated for right and left arms in the patient cohort (mean SVM for each postoperative day/preoperative SVM × 100). The percentage differences in PA between the arms (right and left), were calculated, and the mean percentage difference (± standard deviation) between arms in the population was computed together with a “hand usage ratio” and comparisons between surgical interventions.

The hand usage ratio is defined by the amount of activity in one arm divided by the total combined activities in both arms. The hand ratio of 0.5 for each arm means that the split of arm activity is equal between the right and left arms. A hand ratio increase to more than 0.5 denotes increased activity in that particular arm and vice versa. A right-handed patient will have a right-hand ratio of more than 0.5. In the analysis, bilateral surgery was counted as two independent operations. A parallel study was conducted to establish an understanding of the natural variation between right and left arm activity in 10 healthy volunteers.

### Outcomes

The primary aim of the study was to assess the construct validity of WAMs as an objective tool to measure upper limb function by quantifying the pre- and postoperative arm activity for surgically treated and non-surgically treated (control) sides and by characterizing the physical activities in different types of surgical procedures. The secondary aim was to assess concurrent validity measured by correlating PA with patient-reported upper limb morbidity (DASH questionnaire) and QoL (EQ-5D-5L questionnaire) scores.

### Statistical Analyses

The data were non-normally distributed (Shapiro-Wilk test), so nonparametric statistical tests of significance were performed. The Wilcoxon signed-rank test was used to analyze group differences in longitudinal regain-of-function data. Differences in arm activities between surgical interventions and between surgically treated and control sides were analyzed using the Mann-Whitney *U* test. The PA was determined for each postoperative day (POD) by calculating the percentage of the preoperative SVM level and comparing the level of activities between the surgically treated and control sides as well as between treatment groups, with *p* value lower than 0.05 set as the threshold for statistical significance. Concurrent validity was assessed by calculating Spearman’s correlation coefficient between the PA data and the DASH and EQ-5D-5L questionnaires. The data were analyzed using IBM SPSS Statistics version 26 (IBM, Armonk, New York, NY, USA).

## Results

**Fig. 3:**
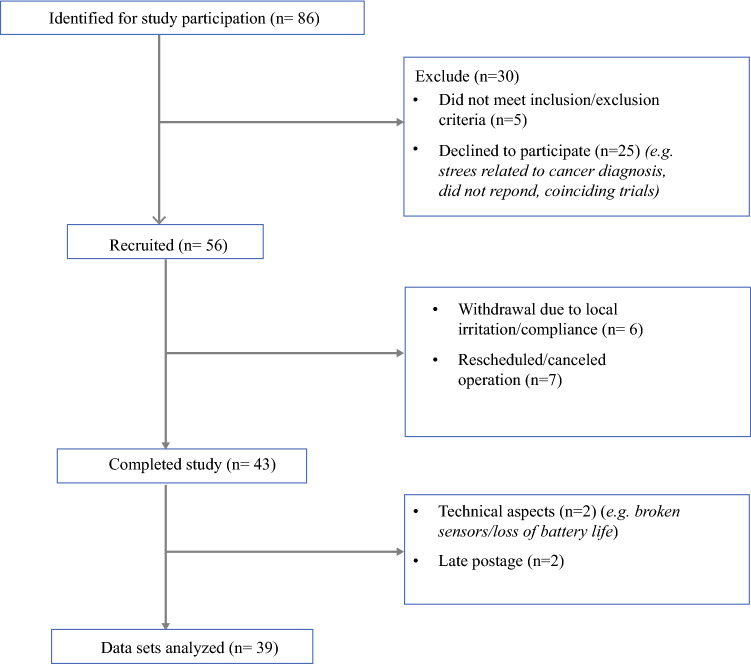
Flow diagram of the study participants. For study participation, 86 patients were identified. The study excluded 30 patients (5 patients did not meet the inclusion and exclusion criteria and 25 patients declined to participate), leaving 56 patients recruited into the study. Six patients withdrew, and seven patients had their operations rescheduled/canceled, leaving 43 patients who completed the study. Of the 43 patients who completed the study, 39 datasets were available for analysis due to technical issues (*n* = 2) and late postage (*n* = 2)

Between April 2019 and December 2020, 86 patients (Fig. 3) treated at Imperial College Healthcare NHS Trust were identified from medical records as potentially eligible. The study was halted from March to July 2020 due to the COVID-19 pandemic. The study excluded 5 patients who did not meet the inclusion and exclusion criteria, 25 patients who declined to participate for various reasons (e.g., stress related to cancer diagnosis, no response, coinciding trials), and 7 patients who had their surgeries rescheduled or canceled. After consideration of other factors including late postage (*n* = 2), technical aspects (battery life, broken sensors) (*n* = 2), and personal factors leading to withdrawal (i.e., local irritation, compliance) (*n* = 6), 39 patient datasets were available for analysis (Table 1). None of the patients included in our study cohort had other relevant history including trauma and shoulder range of movement issues.

**Table 1:** Baseline demographics of the study population

Characteristics	Total (*n* = 39) *n* (%)
Mean age (years)	55 ± 13.2
Sex ratio (M:F)	1:38
Comorbidities	28 (71.8)
Mean BMI (kg/m^2^)	27.9 ± 6.7
Handedness ratio (R:L:BL)	37:1:1
Race/ethnicity White British White Irish White and any other white background Asian and any other Asian background Black or black British African Black or black British Caribbean Black or any other black background Mixed: white and black Caribbean Other: any other ethnic group Other: not stated	9 (23.1) 2 (5.1) 2 (5.1) 3 (7.7) 3 (7.7) 1 (2.6) 1 (2.6) 1 (2.6) 4 (10.3) 13 (33.3)
*Clinical and pathological characteristics*	
Stage of cancer 0 1 2A & B 3A–4 Risk-reducing Benign	7 (17.9) 11 (28.2) 11 (28.2) 6 (15.4) 1 (2.6) 3 (7.7)
Cancer type DCIS Invasive ductal carcinoma Invasive lobular carcinoma Invasive mucinous carcinoma Invasive micropapillary carcinoma BRCA carrier Benign	6 (15.4) 23 (59) 4 (10.3) 1 (2.6) 1 (2.6) 1 (2.6) 3 (7.7)
Previous breast surgery	8 (20.5)
Patients with drain	21 (53.8)
Drain *in situ* in axilla/breast (mean days)	8.6 ± 5.3
Length of stay (days)	1.7 ± 2.1
Advice about exercise	39 (100)
Compliance with analgesia	39 (100)
*Surgical treatment*	
Type of breast surgery^a^ Partial mastectomy Mastectomy IBR Implant-based DIEP TUG TRAM	13 (33.3) 25 (64.1) 12 (30.8) 3 (7.7) 6 (15.4) 1 (2.6) 2 (5.1)
Type of axillary surgery SLNB ALND	16 (41) 14 (35.9)
Operation laterality ratio (R:L:BL)	22:15:2
Neoadjuvant therapy Radiotherapy Chemotherapy Hormone therapy	1 (2.6) 8 (20.5) 6 (15.4)
Adjuvant therapy whilst wearing WAMs Radiotherapy Chemotherapy Hormone therapy	0 0 8 (20.5)

BMI, body mass index; R, right; L, left, BL, bilateral; DCIS, ductal carcinoma *in situ*; IBR, immediate breast reconstruction; DIEP, deep inferior epigastric perforators; TUG, transverse upper gracilis; TRAM, transverse rectus abdominus muscle; SLNB, sentinel lymph node biopsy; ALND, axillary lymph node dissection; WAMs, wearable activity monitors

Means presented with standard deviations, and numbers presented with a whole number percentage of the total population.

^a^Total percentages (%) will not equal 100 because the patients will have a combination of different breast surgeries. The patients who had immediate breast reconstruction surgery also would have a mastectomy.

### Adherence to Wearable Activity Monitors (WAMs)

From all the patients in the study, 546 (39 patients × 14 days) data points in the postoperative period could have been captured. Out of 546 data points, only 59 were not captured, the majority of which were concentrated around days 12 to 14 after surgery. Therefore, the overall adherence to the WAM application protocol was 89.2%.

### Main Findings

#### Regain of Function: Postoperative Physical Activity on the Surgically Treated Side

**Fig. 4:**
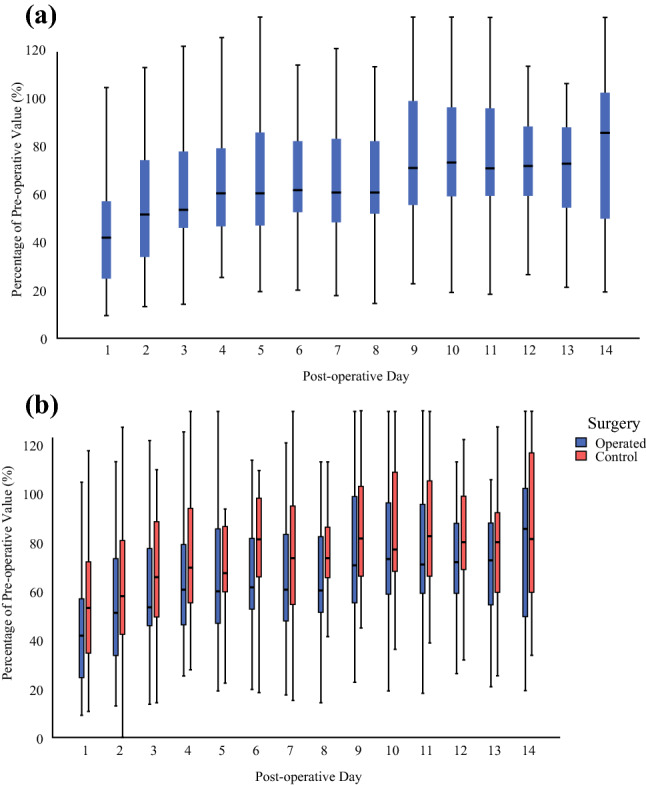
**a** All the surgically treated sides: percentage of preoperative value (%) across a 14-day postoperative period. Physical activity (PA) dropped postoperatively from 100% to 45.3%, and regain of function was gradually observed on the surgically treated side through the increase in PA as a percentage of preoperative levels during the postoperative period. The greatest PA increase (mean PA, 45.3% vs. 56%; *p* < 0.05) was observed between days 1 and 2. The recovery plateau was identified on day 7 (64.7% ± 27.9%), which was the point at which no subsequent significant increase in activity was observed (from day 7 to day 14). **b** Disparity in recovery between the surgically treated arm and control arm movement activity during 2 weeks postoperatively. N.B. (nota bene): All the patients were right-handed except for 1 left-handed and 1 ambidextrous (22 right, 15 left, and 2 bilateral operations). Overall, greater regain of function, represented by greater activity, was observed in the control (non-surgically treated) side compared with the surgically treated side after week 1 (mean PA, 75.8% vs. 62.3%; *p* < 0.0005) and week 2 (mean PA, 91.6% vs. 77.4%; *p* < 0.005). The pattern was maintained throughout for postoperative days 1 to 3 (mean PA, 66.4% vs. 55.3%; *p* < 0.05), days 4 to 6 (mean PA, 83.7% vs. 68.5%; *p* < 0.005), days 7 to 9 (mean PA, 86.4% vs. 71.4%; *p* < 0.05), and days 10 to 12 (mean PA, 91.8% vs. 76.9%; *p* < 0.05). Intergroup comparison between the surgically treated and control sides during the 2 weeks demonstrated a statistically significant difference (*p* < 0.005), with a mean disparity of 13.9% ± 2.3%) between the sides.

 Physical activity dropped postoperatively from 100% to 45.3%, and regain of function was gradually observed on the surgically treated side through the increase in PA as a percentage of preoperative levels during the postoperative period. The greatest PA increase (mean PA, 45.3% vs. 56%; *p* < 0.05) was observed between PODs 1 and 2 (Fig. 4a). The recovery plateau (64.7% ± 27.9%), the point at which no subsequent significant increase in activity was observed (from day 7 to day 14) was identified on day 7.

#### Surgically Treated Side Versus Control Side Activity

Overall, greater regain of function, represented by greater activity, was observed on the control side (non-surgically treated) than on the surgically treated side after week 1 (mean PA, 75.8% vs. 62.3%; *p* < 0.0005) and week 2 (mean PA, 91.6% vs. 77.4%; *p* < 0.005) (Fig. 4b). The pattern was maintained throughout for PODs 1 to 3 (mean PA, 66.4% vs. 55.3%; *p* < 0.05), PODs 4 to 6 (mean PA, 83.7% vs. 68.5%; *p* < 0.005), PODs 7 to 9 (mean PA, 86.4% vs. 71.4%; *p* < 0.05), and PODs 10 to 12 (mean PA, 91.8% vs. 76.9%; *p* < 0.05). Intergroup comparison between the surgically treated and control sides during the 2 weeks demonstrated a statistically significant difference (*p* < 0.005), with a mean disparity of 13.9% ± 2.3% between the two sides. This is higher than the data from 10 healthy volunteers, which demonstrated an average differential activity of 8% ± 5.2%, indicating iatrogenic morbidity from surgery.

#### Hand Usage Ratio and Handedness

**Fig. 5:**
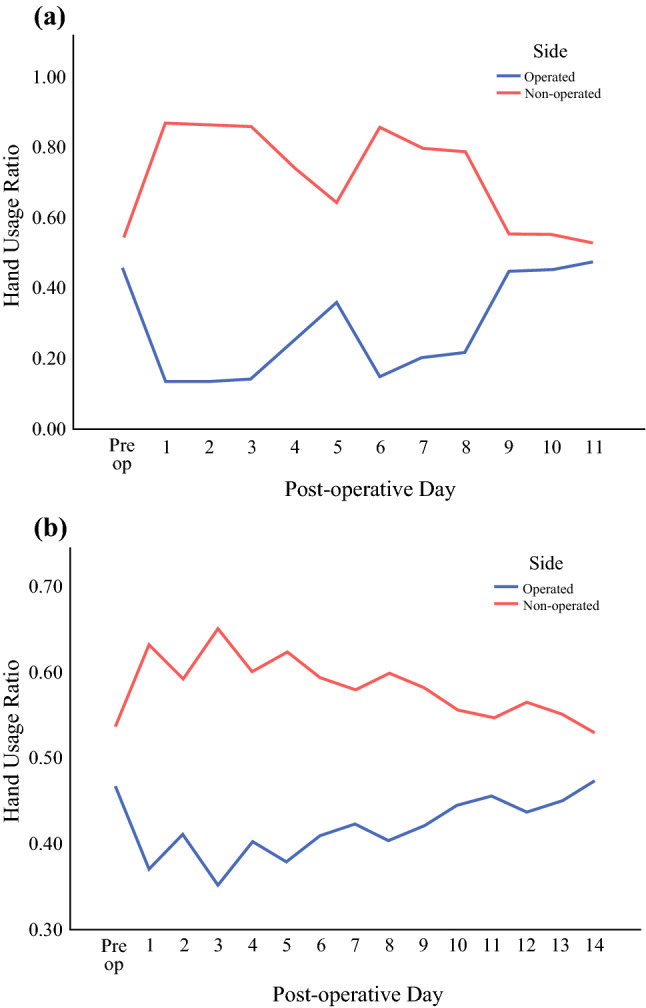
**a** Patient A (right-hand dominant), who underwent left partial mastectomy and SLNB, had greater right-hand usage (0.540) preoperatively. The right-hand usage (non-surgically treated side) increased postoperatively up to 0.867 and gradually returned to the preoperative hand usage level on day 11. **b** Patient B (right-hand dominant), who underwent left  ALND had a preoperative hand ratio of 0.533 increased right-hand usage (non-surgically treated side) postoperatively up to a ratio of 0.648 and returned to preoperative hand usage level on day 14.

The majority of the patients were right-handed, with 1 left-handed patient and 1 ambidextrous patient. Figure 5a illustrates the hand usage ratio of a patient who was right-handed and had a left partial mastectomy and SLNB. Patient A, who was right hand-dominant had a higher right-hand usage (0.540) preoperatively, as depicted in Fig. 5a. The right-hand usage (non-surgically treated side) increased postoperatively up to 0.867 and gradually returned to the preoperative hand usage level on POD 11.

**Fig. 6:**
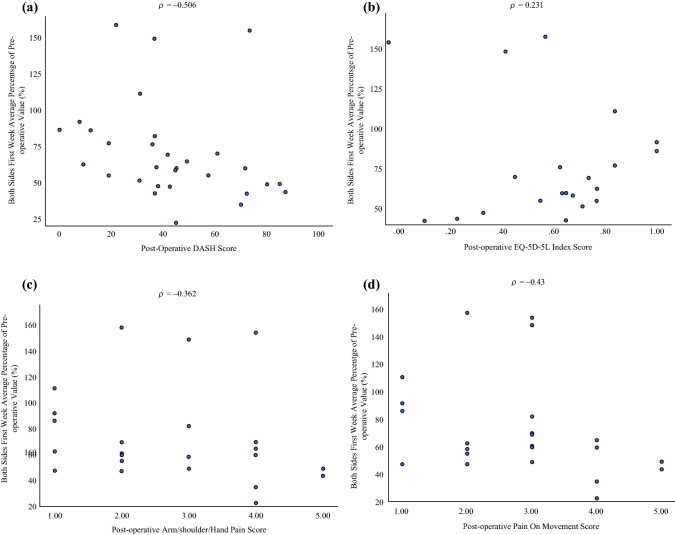
**a** Spearman correlation test between the postoperative Disability of the Arm, Shoulder, and Hand (DASH) score and the activity data measured by wearable activity monitors (WAMs). The postoperative DASH scores were found to have a moderate negative correlation with the activity data (*R* = −0.506; *p* < 0.05). **b** Spearman correlation test between the postoperative EQ-5D-5L score and the activity data measured by WAMs. The EQ-5D-5L scores had a weak correlation with the physical activity (PA) (*R* = 0.231; *p* = 0.313). **c** Spearman correlation test between the postoperative pain score and the activity data. The pain score of the arm, hand, and shoulder showed a weak negative correlation with the activity data (*R* = −0.362; *p* = 0.082). **d** Spearman correlation test between the postoperative score for performance of a specific activity and the activity data measured by WAMs. The postoperative pain score of the arm, hand, and shoulder in performance of a specific activity showed a moderate negative correlation with the physical activities measured by WAMs (*R* = −0.43; *p* < 0.05).

Similarly, patient B (Fig. 5b), who was right-handed with a preoperative hand ratio of 0.533 and had a left ALND increased right-hand usage (non-surgically treated side) postoperatively up to a ratio of 0.648 and returned to preoperative hand usage level on POD 14. Overall, the hand usage of the surgically treated side was reduced from 0.513 to 0.472 and did not return to baseline even on POD 14.

#### Comparison of Surgery Types and the Presence of Drains

Subgroup analyses were performed to compare SLNB with ALND, mastectomy alone with deep inferior epigastric perforators (DIEP) flap reconstruction, and the presence of drains with their absence. The patients receiving ALND (*n* = 14) demonstrated lower PA than the SLNB patients (*n* = 16) after week 1 (mean PA, 58.4% vs. 63.2%; *p* = 0.165) and week 2 (mean PA, 68.5% vs. 75.1%; *p* < 0.05). Compared with mastectomy alone (*n* = 12), significantly lower PA was observed in DIEP reconstruction (*n* = 8) across PODs 1 to 3 (mean PA, 62.5% vs. 44.1%; *p* < 0.05) and after week 1 (mean PA, 66.9% vs. 56.9%; *p* < 0.05). After week 1, the ALND patients who had drains *in situ* were observed to have significantly less PA (57%) than those without drains (64%) (*p* < 0.05).

#### Correlation with DASH and EQ-5D Questionnaires

Comparisons were made with postoperative activity for week 1 (activity in both arms). The postoperative DASH scores were found to have a moderate negative correlation with activity across week 1 (*R* = −0.506; *p* < 0.05; Fig. 6a). The EQ-5D-5L scores had a weak correlation with the PA (*R* = 0.231; *p* = 0.313; Fig. 6b).

#### Correlation with Pain Score

The Spearman correlation test identified a weak negative correlation between upper limb pain scores (*R* = −0.362; *p* = 0.082) and activity data (Fig. 6c). Additionally, the postoperative pain scores for performance of a specific activity showed a moderate negative correlation with physical activities measured by WAMs (*R* = −0.43; *p* < 0.05; Fig. 6d).

## Discussion

This is the first feasibility study to adopt WAMs for objective measurement of upper limb PA after breast and axillary surgery. In this study, WAMs allowed for quantification of pre- and postoperative activity to characterize the recovery curve, measure the disparity between surgically treated and non-surgically treated sides and to compare different types of breast surgery. The results highlighted a significant decrease in arm activity on the surgically treated side and a gradual regain of function between PODs 1 and 14, with the greatest statistical increase in activity occurring in the first 7 PODs. The non-surgically treated side remained more active than the surgically treated side throughout the 2-week postoperative period. Furthermore, the hand usage ratio did not return to normal even 2 weeks after surgery, suggesting that the effects of surgery on the surgically treated side may be beyond the study period, consistent with previous reports.^17^,^19^,^39^,^40^

Postoperatively, arm activity differed significantly between surgically treated side and control side, and it was considerably more than the natural variation seen in the healthy cohort. In comparison with the preoperative data, the hand usage ratio of the non-surgically treated side increased, mirrored by a decrease in the usage of the surgically treated side after surgery, and the ratio did not return to baseline by POD 14. This suggested that the observed variation was unlikely to be solely due to handedness because the system was calibrated to hand usage preoperatively.

There are objective physical assessments of upper limb morbidity among breast cancer survivors such as tape measurement/arm volume and goniometry, but they suffer from variability in measurement protocols and are difficult to perform in clinical settings, time-consuming,^19^,^41^ and operator-dependent.^41^ The effects of hand dominance in relation to side of treatment in assessment of physical function are rarely taken into account when these tools are used.^42^ On the other hand, WAMs allow a standard objective measurement in free-living conditions, calibration of hand dominance, and ease of use. Moreover, WAMs are acceptable to clinicians and patients.^43^–^45^

One of the most common and long-term morbidities associated with breast cancer treatment is lymphedema.^19^ The assessment of lymphedema is complex, and there are many factors that should be considered.^46^ It is important to note that the pathologies of lymphedema may be reflected by arm movement and function. As technological adjuncts, WAMs can be used to assess upper limb activity and function of patients with lymphedema as well as other types of upper limb impairment related to breast cancer treatment such as reduced range of motion, reduced muscle strength, and pain.

In this study, activity levels correlated well with the functional (DASH) questionnaires, implying the concurrent validity of WAMs. Although DASH questionnaires may provide useful information on recovery, the measurements become invalid if used daily due to recall bias.^21^,^47^–^50^ Interestingly, the QoL (EQ-5D-5L) questionnaire scores showed a weak correlation with the activities measured by the WAMs. Our finding is similar to that of Hayes et al.,^42^ whose study also identified a limited correlation between objective and subjective measures.

Taken together, these findings suggest that WAMs and EQ-5D-5L measure different aspects of upper limb dysfunction, and this needs to be considered when an assessment tool is designed. Lymphedema also has greater morbidity than the mere limitation of movement, which could probably explain why no good correlation exists between WAMs and the EQ-5D-5L score. Apart from mobility, self-care, and usual activities assessment, pain/discomfort and anxiety/depression also were included in the EQ-5D-5L survey. Combining objective sensor data with patient-reported outcome such as pain scores may be a solution.^46^,^51^

All the patients in the current study received pain medication. Based on the medical records and patient interviews, all were compliant with their medications. There was a moderate negative correlation between the pain score when performing a specific activity and the WAMs measurement of the activity, suggesting that the upper limb activity could be partly influenced by pain.

It is important to establish a multi-disciplinary approach and work with physiotherapists, engineers, and patients to develop an assessment or screening tool (combination of objective WAMs data and patient-reported outcome [e.g., pain score]) to provide a holistic assessment of breast cancer survivors. Activity monitoring is the only continuous measure of recovery available, which might facilitate real-time patient and clinician feedback. The findings of other studies that incorporated measures such as DASH scores and range-of-motion measurement correlate with our findings of greatest functional morbidity in the initial postoperative period, particularly at week 1, and the initiation of the recovery phase seeming to occur soon after, with the majority of patients reaching the preoperative baseline at 1 month.^52^,^53^

Consultant surgeons and physiotherapists in our unit experienced in using WAMs were confident that this measure of movement would be a reflection of arm function and therefore a surrogate for any factors affecting movement such as stiffness, strength, pain, and swelling, thus reflecting face validity. Construct validity has been demonstrated because the trend of postoperative physical morbidity is as expected (i.e., postoperative decline followed by a gradual return to baseline). The PA data also seem able to differentiate between different surgical procedures, further supporting this claim.

Subgroup analysis demonstrated a trend toward a greater reduction of postoperative activity in the ALND cohort compared with the SLNB cohort in concordance with anecdotal evidence in the literature.^4^,^9^ Similarly, the subgroup analysis of the DIEP patients demonstrated a greater reduction in postoperative activities compared with the patients who had mastectomy alone, showing the iatrogenic morbidity associated with breast reconstruction. Previous studies have shown that autologous breast reconstruction surgery can cause a reduction in strength, shoulder mobility, and function,^54^–^58^ and therefore may adversely affect patients’ activities of daily living and their ability to return to work.^54^ Using the current methodology, WAMs may have a role in answering specific questions about morbidity associated with different surgical procedures and thereby facilitate patients and clinicians in decision-making.

A further application of the technology is to support feedback-enabled prehabilitation and rehabilitation. This may be achieved, with future studies expanding on these data in conjunction with input from engineering specialists and physiotherapists. Postoperative activity data act to keep track of personalized activity goals, such as those already implemented in many enhanced recovery after surgery (ERAS) protocols,^59^ that may empower and motivate patients to take control of their care. Continuation of data collection in the home environment may provide early indicators of complications and make health care professionals aware of patients needing further support, thereby improving their postoperative outcomes.^60^,^61^

It is important to note that the wrist-worn triaxial accelerometers have been validated to measure upper limb activities in previous studies.^35^,^37^,^62^,^63^ Furthermore, wrist-worn triaxial accelerometers are able to recognize specific upper limb movements (e.g., to place a cup on kitchen surface, fetch a kettle, have a drink, retrieve biscuits from a drawer, wipe a table, vacuum) in stroke patients and healthy volunteers.^64^,^65^ However, differentiating purposeful and non-purposeful movement (i.e., upper limb movement to do a specific task vs. upper limb swing during gait) is challenging and may overestimate the upper limb movement.^66^ One way to address this challenge is by quantifying the upper limb movement as a ratio,^62^,^66^ which we have incorporated into the current analysis. In addition, WAMs may underestimate the upper limb movement because they may not capture fine finger movements.^67^ However, the AX3 Axivity (Newcastle upon Tyne, UK) sensor that we used in our study can be calibrated to detect fine movements such as painting and writing.^68^ Further refinement of this technology in the future will help to further improve the accuracy of activity recognition and detection of the upper limb activities.

This study had several limitations including selection bias in favor of more technologically inclined younger patients and those who speak English as a first language; attrition bias that may have occurred due to the withdrawal of more patients with arm problems, complications, readmission, or discomfort while wearing the WAMs; possible confounding factors such as compliance with analgesia, anesthesia effect, adjuvant therapies, and exercise advice as well as natural variety in habitual upper limb movements and handedness; small numbers of patients in subgroup analyses; and device malfunction.

To mitigate these limitations, interpreters were used when available as well as the use of WAMs requiring minimal patient interaction. The baseline characteristics of the patients who withdrew or could not be included due to broken sensors or delays/cancellations after recruitment (*n* = 17) were demographically similar to those of the study patients, limiting the attrition bias. Furthermore, data were collected from healthy subjects for calculation of the normal variation in hand movements. Preoperative activity data were collected, and the hand usage ratio throughout the pre- and postoperative periods was analyzed to allow individualized calibration of postoperative measurements, limiting the effect of handedness. Recruiting patients who had non-breast cancer surgery in future study would better enable control for the general effects of pain and anesthesia on functional recovery.

Attempts to collect data were unsuccessful in 5% of cases due to device error. In the future, we will avoid possible malfunction by completing regular checks of the WAMs before giving them to patients. Further improvement in the technology and design of the WAMs are welcomed to reduce malfunction, improve activity recognition and detection, and improve wearability and adherence. We collected feedback from our patients and made a small adjustment (e.g., adding an extra loop on the bracelets) to the WAMs during our study to improve adherence and wearability. We also are working with patients and a group of engineers to improve the technology for future studies.

In the future, data collected at different time points (3, 6, 12, and 18 months postoperatively) may allow more exact quantification of temporal return to baseline activity. Using a combination approach, such as using the WAM data and a patient-reported outcome (e.g., pain score), an assessment or screening tool could be developed that would be able to capture upper limb impairment and provide a reliable, objective assessment of upper limb function inexpensively and with minimal technical support required.

## Conclusion

In conclusion, this study demonstrated the feasibility and validity of WAMs toward longitudinal objective monitoring of limb recovery after breast and axillary surgery. This facilitates more objective profiling of the morbidity from breast cancer treatments. The results highlight a decline in arm function on the surgically treated side and a gradual recovery that does not return to baseline even 2 weeks after surgery. The current work will improve the protocols and usability of WAMs for future studies with more defined scientific hypotheses. Quantifying or characterizing the morbidity of different treatments may help the clinician and patient to choose the most suitable method, particularly where oncologic outcomes are equivocal. It also may lead to a technological solution for enhanced assessment of upper limb functional outcomes after surgery as well as developing a platform for novel personalized prehabilitation and rehabilitation strategies.

## Supplementary Information

Below is the link to the electronic supplementary material.

Supplementary file1 (DOCX 274 kb)
