# Supplementary material for: Objective Assessment of Postoperative Morbidity After Breast Cancer Treatments with Wearable Activity Monitors: The “BRACELET” Study
Source: Ann Surg Oncol. 2021 Jul 26;28(10):5597–609. doi: 10.1245/s10434-021-10458-4 (PMC8312212; doi:10.1245/s10434-021-10458-4)
Supplement: Supplementary file 1 — Supplementary file1 (DOCX 274 kb) [file 10434_2021_10458_MOESM1_ESM.docx]

**Supplementary Figure 1**


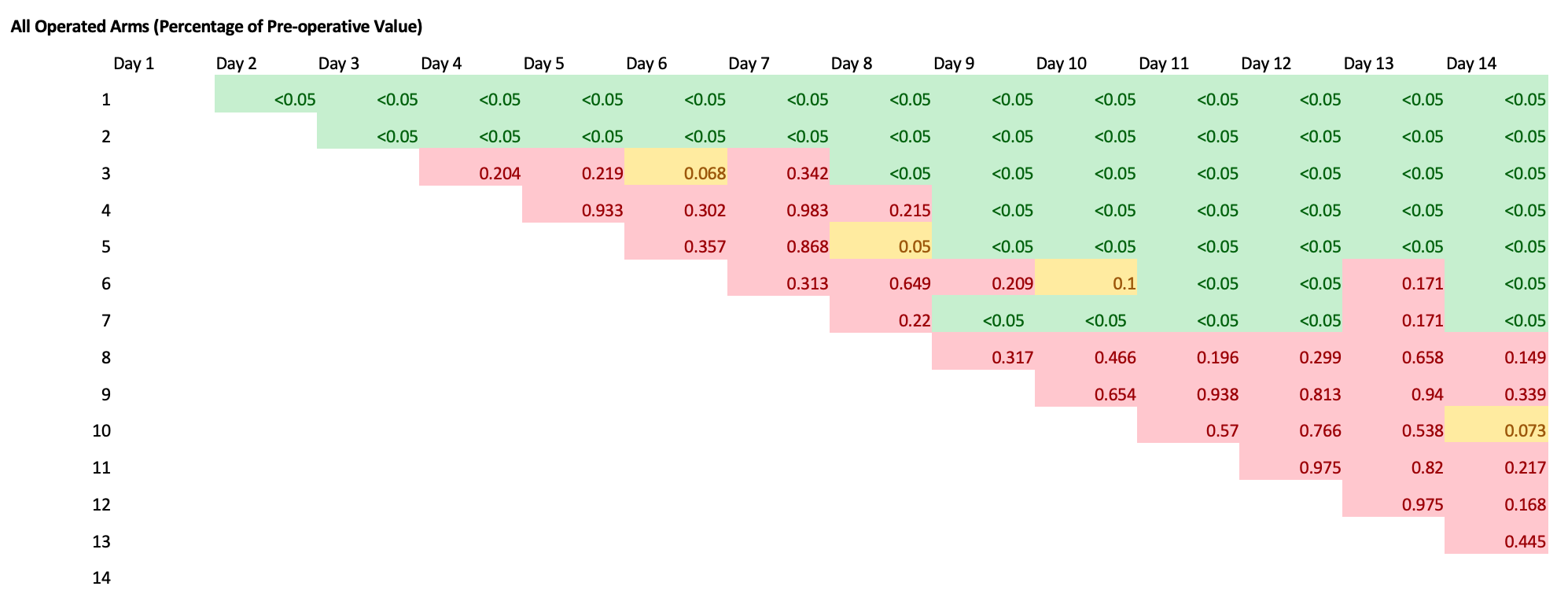


**Figure 1:** Wilcoxon signed-rank test for operated side activity pairwise comparisons were conducted for every combination of post-operative day to determine the recovery plateau. Statistical significance reflected by ‘traffic light’ color scheme from p<0.05 as green to p = 1 as red. The recovery plateau was identified on day 7, 64.7%, (SD: 27.9), which was the point at which no subsequent significant increase in activity was observed from day 7 to 14.

**Supplementary Table 2**

| Day | Operated side  (ratio) | Non-operated side (ratio) |
| --- | --- | --- |
| Pre-op | 0.513 | 0.487 |
| 1 | 0.472 | 0.528 |
| 2 | 0.472 | 0.528 |
| 3 | 0.478 | 0.522 |
| 4 | 0.478 | 0.522 |
| 5 | 0.474 | 0.526 |
| 6 | 0.474 | 0.526 |
| 7 | 0.478 | 0.522 |
| 8 | 0.471 | 0.529 |
| 9 | 0.475 | 0.525 |
| 10 | 0.482 | 0.518 |
| 11 | 0.478 | 0.522 |
| 12 | 0.478 | 0.522 |
| 13 | 0.472 | 0.528 |
| 14 | 0.477 | 0.523 |

**Table 2:** The ratio for operated side and non-operated side for all patients included in the study (n=39)

**Supplementary Figure 3**

**
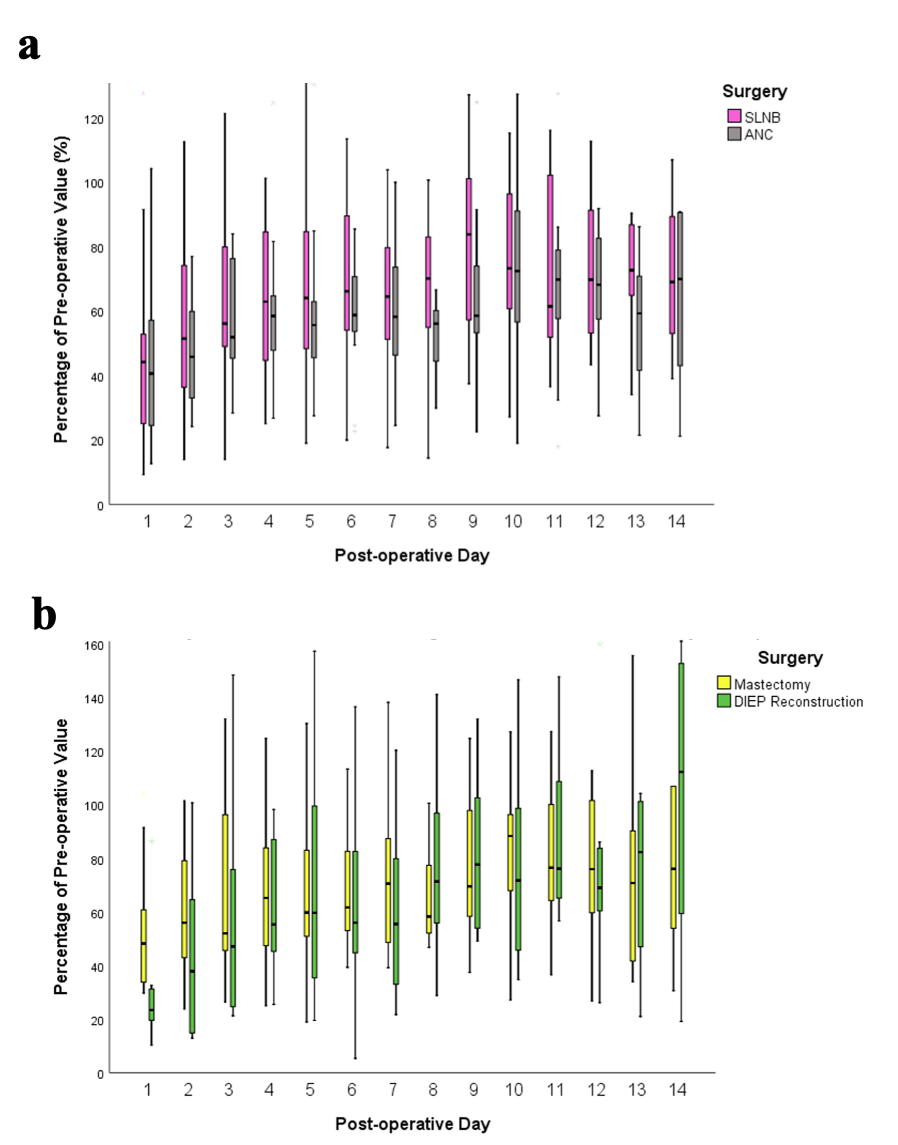
**

**Figure 3**: **(a)** Sentinel lymph node biopsy (n=16) vs axillary lymph node dissection (n=14) across a 14-day post-operative period. Patients receiving ALND demonstrated lower PA compared to SLNB patients after week 1 (mean PA: 58.4% vs mean PA: 63.2%, p=0.165) and week 2 (mean PA: 68.5% vs mean PA: 75.1%, p<0.05). **(b)** Mastectomy alone (n=12) vs DIEP reconstruction (n=8) across a 14-day post-operative period. Compared to mastectomy alone, significantly lower PA was observed in DIEP reconstruction across days 1-3 (mean PA: 62.5% vs mean PA: 44.1%, p<0.05) and after week 1 (mean PA: 66.9% vs mean PA: 56.9%, p<0.05).
